# Color Perception in Protanomalous Female *Macaca fascicularis*

**DOI:** 10.1177/2041669519846136

**Published:** 2019-04-29

**Authors:** Kanthi A. Widayati, Atsuko Saito, Bambang Suryobroto, Akichika Mikami, Kowa Koida

**Affiliations:** Department of Biology, Bogor Agricultural University, Indonesia; Department of Psychology, Faculty of Human Sciences, Sophia University, Tokyo, Japan; Department of Biology, Bogor Agricultural University, Indonesia; Faculty of Nursing and Rehabilitation, Chubu Gakuin University, Seki, Japan; Electronics-Inspired Interdisciplinary Research Institute, Toyohashi University of Technology, Japan; Department of Computer Science and Engineering, Toyohashi University of Technology, Japan

**Keywords:** color vision, macaque, protanomalous female, L4M5 opsin

## Abstract

Protanomalous females with X chromosome-linked color vision deficiency exhibit mild abnormalities, whereas dichromats show a distinct deficiency in discriminating certain color pairs. Dichromats have an advantage in detecting a textured target when it is camouflaged by red-green colors, owing to their insensitivity to these colors. However, it is not certain whether protanomalous females possess a similar advantage in breaking camouflage. Here, we introduce an animal model of dichromatic macaque monkeys and protanomalous females. We examined whether protanomalous females have the same advantage in breaking color camouflage as shown by dichromatic macaques. We also tested whether they could discriminate a certain color pair that trichromats could, where the dichromats are confused. Our experiments show that protanomalous macaques can break color camouflage, similar to dichromats, and can discriminate colors similarly to trichromats. Protanomalous females are thus thought to have the combined ecological advantages of being both trichromats and dichromats.

## Introduction

The salience of hues that we perceive is facilitated by trichromatic color vision. This vision arises from the possession of three types of opsins in the cone photoreceptors that absorb short (blue)-, middle (green)-, and long (red)-wavelength spectra of visible light with the maximum sensitivities lying near 436, 530, and 564 nm, respectively (Baylor, Nunn, & Schnapf, 1987; Bowmaker, Astell, Hunt, & Mollon, 1991; Merbs & Nathans, 1992).

In humans and in Old World primates that possess routine trichromacy, genes located in autosomes and sex chromosomes encode the opsins. S opsin (blue) is encoded by the *S* gene on an autosome, whereas L (red) and M (green) opsins are encoded by *L* and *M* genes, which reside in a tail-to-head tandem arrangement within the X chromosome (Dulai, Bowmaker, Mollon, & Hunt, 1994; Onishi et al., 2002). The *L* and *M* genes have 98% similarity in nucleotide sequence. The similarity between the nucleotide sequences of *L* and *M* genes predisposes the X chromosome-located tandem array to unequal homologous recombination (Nathans, Piantanida, Eddy, Shows, & Hogness, 1986) in both the intragenic and intergenic regions of the genes. Unequal intragenic or intergenic recombination can cause defects in the original *L* and *M* opsin genes. A male subject lacking one opsin gene in the X chromosome becomes a dichromat and cannot discriminate certain combinations of colors that can be perceived by trichromats.

Trichromatic and dichromatic individuals have been shown to exhibit different perceptions of color, which provides particular ecological advantages and disadvantages to each group. Habitual trichromacy enables Old and New World primates to detect either reddish matured fruits or young leaves against green foliage background, which is thought to play an important role in survival (Caine & Mundy, 2000; Dominy & Lucas, 2001; Lucas, Darvell, Lee, Yuen, & Choong, 1998; Melin et al., 2009; Riba-Hernandez, Stoner, & Osorio, 2004; Smith, Buchanan-Smith, Surridge, Osorio, & Mundy, 2003). Trichromacy also plays an important role in discriminating the spectral modulations on the skin of conspecifics, presumably playing a role in discriminating emotional states, socio-sexual signals, and threat displays (Changizi, Zhang, & Shimojo, 2006). On the other hand, congenital dichromats of humans and New World Monkeys perform better under color-camouflaged conditions (Melin et al., 2009; Morgan, Adam, & Mollon, 1992; Saito, Mikami, Hosokawa, & Hasegawa, 2006; Saito et al., 2005).

Males have a higher possibility of color vision abnormalities compared to females, as they have only one X chromosome. About 2% of male humans are dichromats (Sharpe, Stockman, Jagle, & Nathans, 1999) and 5% to 11% male humans possess anomalous color vision; thus, dichromacy and anomalous color vision are not a rare state in humans (Drummond-Borg, Deeb, & Motulsky, 1989). Unlike humans, in nonhuman Old World primates, positive selection of normal trichromacy might put exceptional pressure on other phenotypes (Bowmaker et al., 1991; Jacobs & Deegan, 1999). Genetic surveys were conducted to test gene deletion or the existence of hybrid genes in apes and old world monkeys (Hiwatashi et al., 2011; Onishi et al., 2002; Terao et al., 2005). The frequencies of color vision variants in male chimpanzees and male long-tailed macaques were ∼ 1.7% and ∼ 0.4%, respectively (Onishi et al., 1999; Onishi et al., 2002; Terao et al., 2005). Despite the fact that these frequencies are clearly lower than those in humans, our research group has found three wild-born long-tailed male macaques (*Macaca fascicularis*) possessing a recombinant gene of *L* and *M* in their X chromosomes. As a result, the three male monkeys were dichromats with only one opsin gene in their single X chromosome. The gene in this X chromosome was actually a hybrid allele, comprising exon 1 to exon 4 of the *L* opsin gene and exon 5 to exon 6 of the *M* opsin gene. We designated this allele as *L4M5* (Onishi et al., 1999). The maximum spectral sensitivity of the hybrid opsin differs by only 6 nm from that of the M opsin. Consequently, these macaques exhibit protanopia color vision deficiency. Electroretinogram analyses and behavioral tests also support the finding that the animals were protanopic. The genotypic dichromats showed reduced sensitivity to long-wavelength light compared to trichromatic individuals (Hanazawa et al., 2001) and could not discriminate between human protanopic confusion colors (Koida et al., 2013).

We also found some females of the species carrying *L* and *M* wild-type genes in one X chromosome and a single *L4M5* allele in her other X chromosome (Onishi et al., 2002). In the female, one of the two copies of the X chromosome is randomly inactivated in any given cell and remains inactive throughout the cell lifetime (Lyonization/X inactivation; Lyon, 1962). A female can possess four types of opsins: S, M, L, and L4M5. The *L4M5* allele-carrying females might normally be considered *carriers* of the mutation but, in this article, we tested whether the effect of the mutation is fully masked; thus, we called these females as *protanomalous females* (Kawamura, 2016). Electroretinogram analyses of the protanomalous female showed sensitivity between trichromats and protanopes; the relative sensitivities to 644 nm light in trichromatic, protanomalous, and protanope individuals were measured to be about 4:2:1, respectively (Hanazawa et al., 2001).

Human females possessing the heterozygous color-blind gene usually exhibit trichromacy as they pass a color vision test; however, some psychophysical studies have shown that the these females make more errors in detecting colored numbers in pseudoisochromatic plates compared to trichromats and perform differently in the color mixture task (Cohn, Emmerrich, & Carlson, 1989; Jordan & Mollon, 1997; Konstantakopoulou, Rodriguez-Carmona, & Barbur, 2012; Lang & Good, 2001; Nagy, MacLeod, Heyneman, & Eisner, 1981). Moreover, they show a partial reduction in sensitivity to red light, known as the Schmidt sign (Jordan & Mollon, 1997).

No experimental behavioral study has been conducted on the color perception of the protanomalous female in *M. fascicularis*. To understand the physiological basis of dichromacy using model animals, it is important to know how these protanomalous monkeys differ in color vision performance beforehand. To determine whether protanomalous females have the advantages and disadvantages of both trichromacy and dichromacy, we tested them using two behavioral paradigms: the dichromatic advantage paradigm and the trichromatic advantage paradigm. In the dichromatic advantage paradigm, the object contour is defined by luminance but is masked by confusion-colored chromatic noise. This makes the camouflage invisible and ineffective to dichromats, but effective to trichromats (Saito et al., 2005). As a consequence of the Lyonization process, the protanomalous female has a relatively small population of L cones compared to the other type of cones, whereas normal trichromats have almost an equal number of L and M cones. A relatively large LM ratio might induce a small L-M color signal because subtraction on retinal ganglion cells is thought to be a stochastic event; thus, an equal LM ratio would be optimal for deriving the color signal. On the other hand, the luminance signal is not strongly affected by the class of cones. Thus, the color-camouflaging signal might have a weak impact on the luminance detection task and the protanomalous female may disregard the color camouflage and recognize the luminance contour more easily compared to normal trichromats. In the trichromatic advantage paradigm, the object contour is defined by protanopic confusion colors and is masked by luminance noise. Thus, the targets should be invisible to dichromats but visible to trichromats. We predict that the amount of L cones is enough to discriminate the protanopic confusion colors and render them visible to the protanomalous female.

## Methods

### Subjects

Two adult protanomalous females (named Suri and Kerok), two adult trichromatic (Ucok and Manis), and two adult dichromatic males (Pedas and Pait) of long-tailed macaques participated in these experiments. The same trichromatic and dichromatic male macaques also participated in our previous experiments on the dichromatic advantage paradigm (Saito et al., 2005). These macaques, except Kerok (see later), were caught in the wild in 1998 and 1999 in the Pangandaran National Park of Indonesia and were cared for at the Department of Biology of Bogor Agricultural University with approval from the Ministry of Forestry, Indonesia. We bred them in the laboratory and obtained only one protanomalous female, Kerok. Today, Kerok is the only protanomalous female who has lived entirely in captivity; the other died due to old age. This experiment was conducted from September 2004 until December 2005 in Indonesia and from August to December 2008 in Japan.

The opsin genes were detected in the subjects using molecular methods (Onishi et al., 2002). This gene might be a result of unequal crossing-over between the *L* and *M* genes and is not a part of gene duplication. There were no multiple *L* or *M* genes in our subjects including the L4M5 male and protanomalous female (Onishi et al., 2002). At Bogor Agricultural University, the macaques were kept in group cages with other monkeys and were moved to individual cages (70 × 80 × 90 cm^3^) during the experiment. Kerok was transferred to the Primate Research Institute of Kyoto University in Japan during the experiment. She was reared in an individual cage (70 × 80 × 85 cm^3^) and was tested in the same cage. The experiments were conducted according to the Guide for the Care and Use of Laboratory Animals by the National Institute of Health, USA (1985), and the Guide for the Care and Use of Laboratory Primates by the Primate Research Institute, Kyoto University (1986, 2002). This study also adhered to the American Society of Primatologists principles for the ethical treatment of nonhuman primates.

To circumvent sample limitations, we used repeated trial experiments taken serially in time to measure the subjects’ responses. We first trained the subjects to choose a particular stimulus from an alternative. After that, we tested their inherent color perception by examining their response to particular stimuli (for details, see Experimental Procedure). We assumed that the response between the trials was independent and was not correlated due to learning; thus, we could count the frequency of the response and compare the different frequencies of each phenotype in statistical analysis.

### Experiment 1: Dichromatic Advantage Paradigm

The animals performed two-alternative forced choice tasks in which the target was determined by a luminance texture, which was camouflaged by red-green colored noise.

#### Stimuli

**Figure 1.**
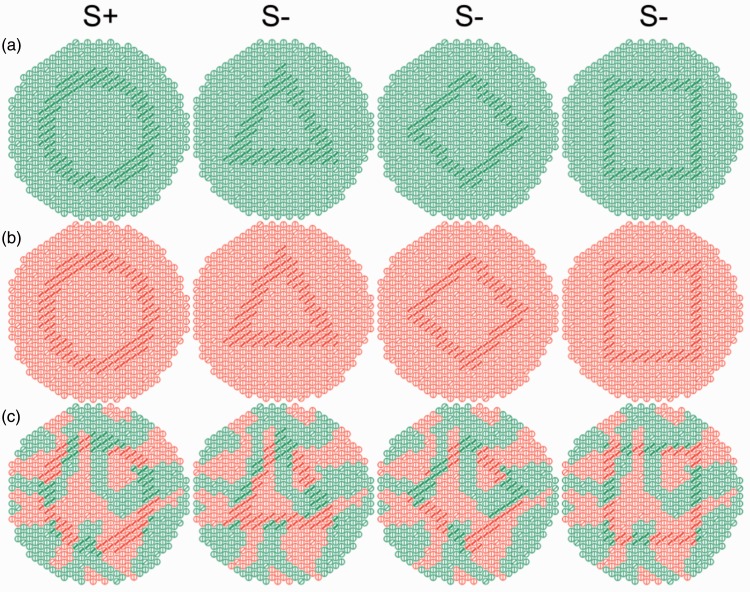
Stimuli in the dichromatic advantage paradigm. Monkeys had to choose the card with the ring (S+) to get a reward when it was paired with a card that contains other patterns (S−). In one trial, we paired the ringed card with one of S−, printed with a triangle, lozenge, or square. These patterns were made using textural elements that differ in orientation and thickness from those of background elements. All colors used for the stimuli are based on protanopic confusion colors (Figure 2(a) and (b)). (a) and (b) Stimuli used as the baseline. (c) Color-camouflaged stimuli; red and green mosaic patches of colors were added so that the target patterns were camouflaged.

**Figure 2.**
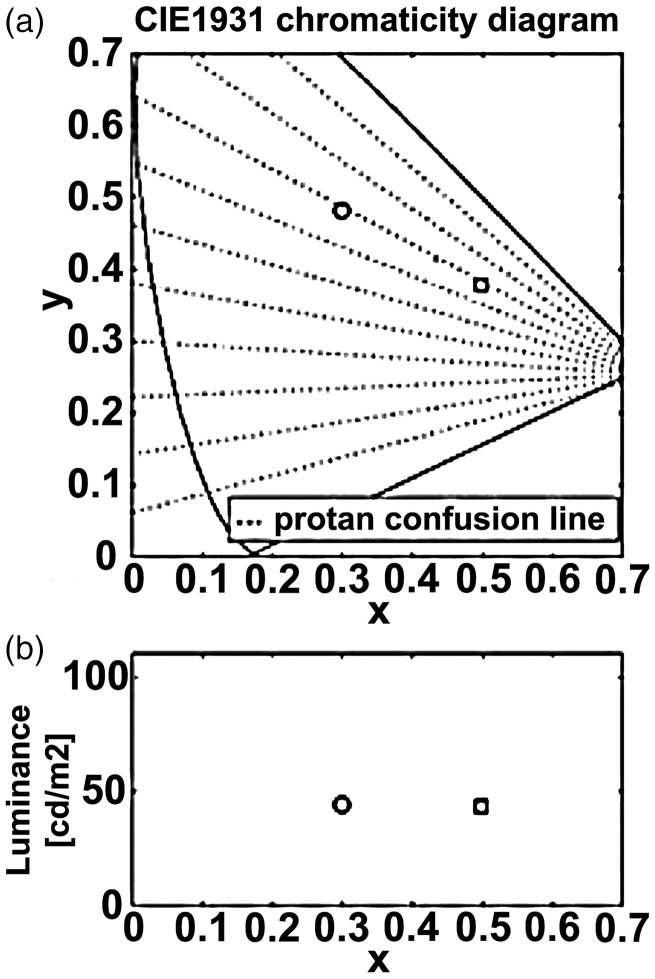
Chromaticity and luminance of color elements in color-camouflaged stimuli. (a) The horizontal and vertical axes show the *x* and *y* values of the CIE1931 chromaticity diagram, respectively. Both reds (squares) and greens (circles) are located along the same protanopic confusion line. (b) The vertical axis shows the luminance and the horizontal axis is the same as in (a).

The stimuli were the same as those in our previous study (Saito et al., 2005). We prepared green (Figure 1(a)) and red (Figure 1(b)) stimulus sets based on the protanopic confusion line in the CIE 1931 chromaticity diagram (open symbols in Figure 2(a)). The color luminance is shown in Figure 2(b). Each stimulus, with 55 mm diameter, was printed on a glossy paper (Kokuyo, KJ-AG1315) using an ink-jet printer (Epson, PM-800C). The experimental room was illuminated with natural light and a conventional fluorescent light. The colors of the stimuli were measured under a fluorescent light (FL20S.D-Edl-D65; Toshiba Light and Technology, Co., Tokyo, Japan) with a Minolta Chroma Meter CS-100. The chromaticity and luminance of the printed images can vary depending on the illumination, and natural light is not static; however, two colors composing the stimuli were always a protanopic confusion pair in the experimental room. This was repeatedly confirmed by dichromatic human observers. Given the fact that the color of the fluorescent light (D65) was similar to that of natural light, we considered that the effect of natural illumination was negligible and that the color stimuli were a protanopic confusion pair.

#### Equipment

A modified version of the Wisconsin General Test Apparatus was used for presenting the stimuli to the macaques. The apparatus used for five subjects at the Bogor Agricultural University comprised a horizontal tray (44 × 30.5 cm^2^) containing two food wells (diameter 4.5 cm) mounted on a portable shelf. These food wells were 21 cm apart from each other center-to-center and were covered with a lid so that the subjects needed to pull the lever to open the lid and uncover the stimuli. The two stimuli were covered with a lid (44 × 15 cm^2^) that was hinged to the tray. The tray was placed in front of an individual test cage. A timer was set to start automatically when the subjects opened the lid by pulling the lever attached to the lid. The timer was set to stop automatically when the subjects touched one of the stimuli. The duration between the start and stop of the timer was defined as the reaction time (Saito et al., 2005).

For Kerok in Kyoto University, we used a similar tray without a lid. When setting up the food reward between trials, an opaque screen was placed between the cage and the experimenter to prevent animals from seeing the process.

#### Experimental procedure

We used an operant conditioning technique to train the subjects to choose one out of two stimuli at every trial. The stimulus pair consisted of a ring-patterned card and another card that contained an alternative pattern. The ring-patterned card (called S+) was a correct stimulus, and when the animal picked up S+, they would receive a food reward. When the animal picked up the other card (the triangle-, lozenge-, and square-patterned, S−), they would not receive a reward. With this conditioning, the reward was expected to motivate the subjects to always choose S+. One experimental session consisted of 20 trials, and two or three training sessions were conducted for an individual per day.

The subjects were first trained with green stimuli (Figure 1(a)). This training continued until the percentage of those who chose S+ reached 90% or higher in three successive sessions. After that, the subjects were trained with red stimuli (Figure 1(b)). When the subjects reached a score of 90% correct choices in three successive sessions, they continued training with pseudo-randomly mixed stimuli, colored either red or green. The subjects had to reach 90% or higher in three successive sessions of these serially mixed stimuli. After meeting the criteria, the test experiments using color-camouflaged stimuli were initiated (Figure 1(c)).

Each test session comprised three probe trials (color-camouflaged stimuli) pseudo-randomly mixed among 17 baseline trials (green or red stimuli). S+ was rewarded in both the probe and baseline trials in the test sessions, which were run 10 times to accumulate 30 probe trials. Data from a test session would be aggregated into the analysis if the percentage of correct responses in its baseline trials were significantly higher than the chance level (50%). This quality control asserts that the subject could truly discriminate S+ from S− in the session. For this experiment, 5 out of 15 sessions were excluded for one trichromatic male. No exclusion was performed for the other animals. We used the binomial statistical test (Conover, 1971) to determine if the subject performances were significantly different from the chance level. We expected different performances in the protanomalous, trichromatic, and dichromatic monkeys in the baseline and probe trials. To compare the proportion of success between the baseline and probe trials, we used the proportion test (Wilson, 1972). For each phenotype group, we measured the response of two animals. Statistical analysis was conducted for each animal as a repeated measurement.

### Experiment 2: Trichromatic Advantage Paradigm

**Figure 3.**
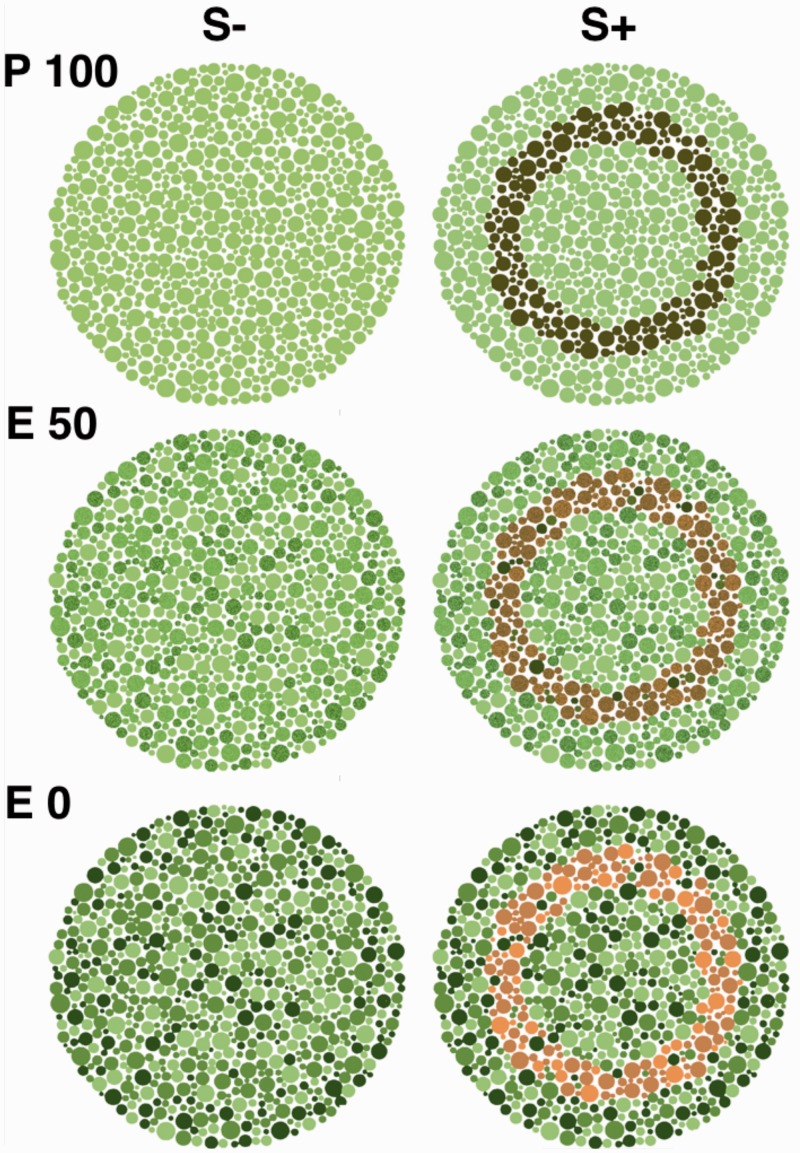
Stimuli in the trichromatic advantage paradigm. Monkeys had to choose the card with a ring (S+) to get a reward when it was paired with a card without a ring (S−). The rings of S+ of E0 were made from colors that cannot be distinguished by protanopic people (Figure 4) and acted as a probe for the color-blind test. P100 and E50 pairs were used in the training sessions. The E50 pair was also used as the baseline stimuli in the test sessions.

Since protanomalous females have S, M, and L opsins in their retina, they should be able to discriminate colors just like the trichromatic observers. To confirm this, we used a modified version of the pseudoisochromatic color-blind test for humans (Saito et al., 2003), and modified the test card so that trichromatic macaques would see the pattern in the form of a ring (E0 set, Figure 3). The animals performed two-alternative forced choice tasks with the stimulus pair S+ and S−. By using the same operant conditioning technique as in Experiment 1 and by arbitrarily defining the card with the ring as the S+ stimulus, we might infer their vision based on their responses to the stimuli. In the rest of this article, we refer to this experiment as the *color-blind test*.

#### Stimuli

**Figure 4.**
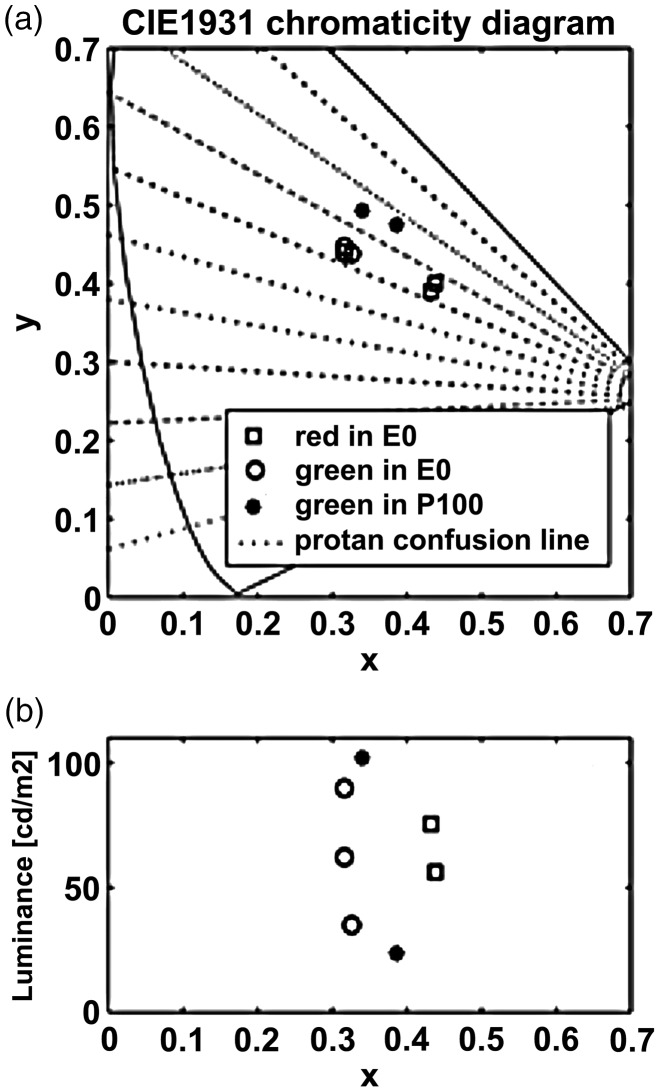
Chromaticity and luminance of color elements in E0 and P100. (a) The horizontal and vertical axes show the *x* and y values of the CIE1931 chromaticity diagram, respectively. Both reds (squares) and greens (circles) are located along the same protanopic confusion line. (b) The vertical axis shows the luminance and the horizontal axis is the same as that in (a).

The stimuli used in the color-blind test were the same as those used in the previous article (Saito et al., 2003). Three stimulus sets, P100, E50, and E0, were used in this experiment (Figure 3). P100 had a target ring determined by luminance contrast, whereas E0 was determined by color contrast (Figure 4). The stimulus set E50 was made by dither mixing the colors of P100 and E0 at a ratio of 50:50. Each panel, 6.0 cm in diameter, was printed on glossy paper (KJ-AG1315, Kokuyo OA) using an ink-jet printer (PM-4000PX, Epson). The experimental room was illuminated by natural light. Stimulus measurements and calibration were the same as those in Experiment 1.

#### Equipment

We used the same apparatus as in Experiment 1.

#### Experimental procedure

As in Experiment 1, two or three sessions of training were conducted for each individual per day. One session comprised 20 trials during training. Using the P100 pair, the subjects were first trained to choose the ring-patterned card (S+) instead of a card without a pattern (S−). After the subjects learned the stimuli, that is, they reached 90% correct choices in three successive sessions, the subjects progressed to the next step of training consisting of mixed trials with P100 and E50 pairs. We used E50 so that the monkeys would get used to the variation in luminance and color. The proportion of E50 trials gradually increased until all trials consisted of only the E50 set. Training with the all-E50 stimulus set was continued until the animals reached 90% correct choices in three successive sessions.

After completing this criterion, test sessions were conducted for each individual. Each test session consisted of 17 baseline trials with the E50 stimulus and 1 probe trial with the E0 stimulus set. The probe trials were serially mixed with baseline trials in a pseudo-random manner. Depending on the monkey, the test sessions were run 10 to 40 times to obtain 10 to 40 probe trials in total. As in Experiment 1, data from the test sessions were analyzed only when the percentage of correct responses in the baseline trials of the test session was significantly higher than the chance level. No exclusion of sessions was performed for all subjects in this experiment. Statistical tests were the same as those used in Experiment 1.

## Results

### Experiment 1: Dichromatic Advantage Paradigm

**Figure 5.**
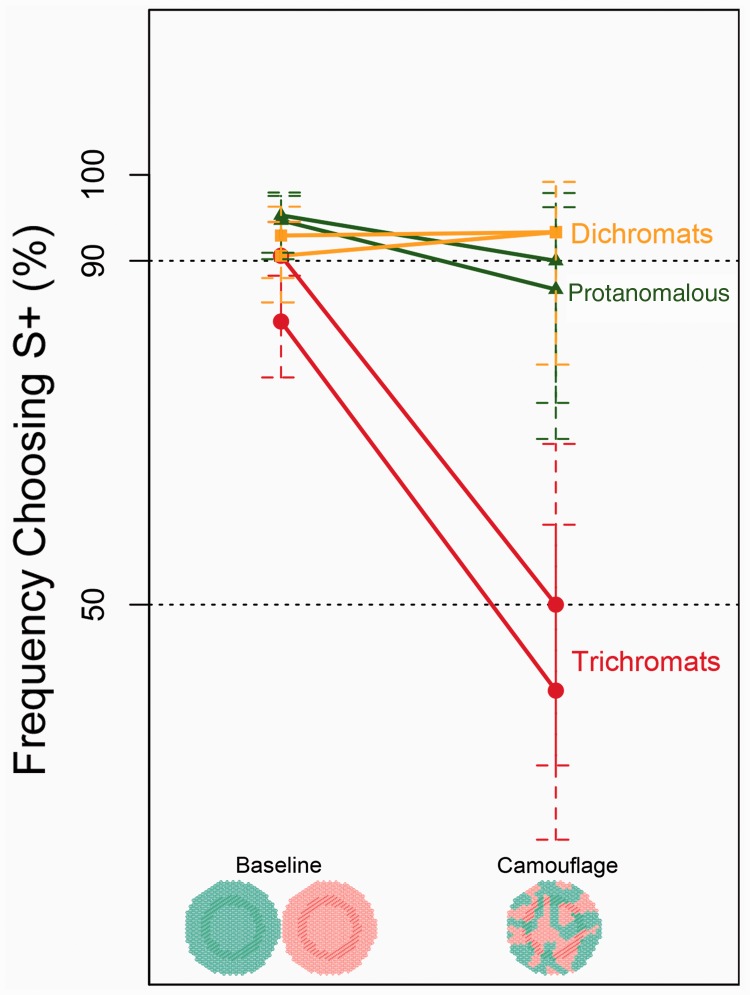
Results of the dichromatic advantage paradigm. Each dot represents the performance of an individual monkey in both the baseline and color-camouflaged conditions. The vertical lines passing through the dots show 95% confidence intervals in the binomial test. The performance levels of the dichromatic and protanomalous monkeys at camouflage were not significantly different from the baseline trials (baseline: *n* = 170, camouflage: *n* = 30 for each subject, *p* = .89 and *p* = 1 for dichromats, *p* = .21 and *p* = .46 for protanomalous by the proportion test). In contrast, the trichromatic monkeys reached only around 50% correct choices in the color-camouflaged condition, being significantly lower than that in the baseline trials (*n* = 170 for baseline and *n* = 30 for camouflage for each subject, *p* = 1.5 × 10^−6^ and *p* = 7.0 × 10^−8^ by a proportion test).

Figure 5 shows the results of the color-camouflage test. The data reported here for the dichromatic males and trichromatic males in this experiment are the same as those reported by Saito et al. (2005). The protanomalous female macaques could discriminate the patterns in the baseline trials for more than 90% during the test sessions. They also discriminated the patterns in the probe trials of color-camouflaged cards for about 90%. These two performance levels were not significantly different for each animal (proportion test, *p* = .21,* p* = .46). These results were similar to those of the dichromatic macaques and indicated that the protanomalous and dichromatic macaques were not affected by the color-camouflaged condition. In contrast, although the trichromatic macaques could choose S+ in the baseline trials for about 90%, they chose it randomly in the probe trials. The performance was not significantly different from that in the chance level (binomial test, *p* = .4, *p* = 1) and was significantly different from that in the baseline trials (proportion test, *p* = 1.5 × 10^−6^, *p* = 7 × 10^−8^), indicating that they failed to discriminate between S+ and S− in the color-camouflaged condition (Saito et al., 2005). Thus, the results indicate that the protanomalous female had the same ability as dichromatic macaques to break the color-camouflaged condition, whereas the trichromatic animals failed.

We collected the reaction time data for only five monkeys (two trichromatic, two dichromatic, and one protanomalous female). The average reaction time of trichromatic monkeys in choosing the camouflage stimuli was longer (Ucok: 1,728 ms; Manis: 2,456 ms) than that in choosing the baseline stimuli (Ucok: 1,474 ms; Manis: 2,138 ms), though it was not statistically significant for one monkey (*t* test, *p* = .008 for Ucok, *p* = .18 for Manis). The average reaction time of dichromats was the same as the camouflage condition (Pedas: 1,782 ms; Pait: 1,889 ms) and baseline condition (Pedas: 1,837 ms; Pait: 1,865 ms, *t* test, *p* > .05 for both animals; Saito et al., 2005). The protanomalous female also did not differ in reaction time between the baseline (1,905 ms) and camouflage conditions (1,989 ms, *t* test, *p* > .05). This result emphasizes that protanomalous female monkeys showed the same performance as dichromat monkeys in choosing the camouflage stimuli.

### Experiment 2: Trichromatic Advantage Paradigm

**Figure 6.**
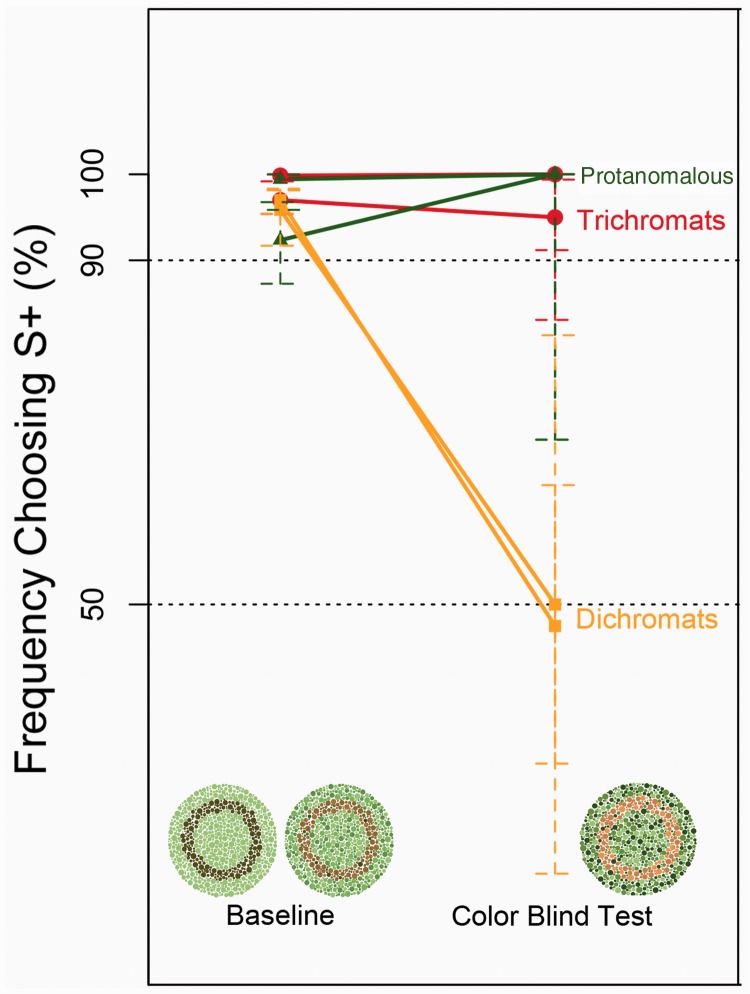
Results of the trichromatic advantage paradigm. Each dot represents the performance of an individual monkey in both the baseline and color-blind test. The performance levels of trichromatic and protanomalous monkeys in the color-blind test were not significantly different from the baseline trials (baseline: *n* = 170–680, color-blind test: *n* = 10–40, *p* = .81 and *p* = 1 for trichromats, *p* = .78 and *p* = 1 for protanomalous by the proportion test). In contrast, the dichromatic monkeys reached only around 50% in the probe trials, which was significantly lower than that in the baseline trials (baseline: *n* = 170–680, color-blind test: *n* = 10–40, *p = *5.7 × 10^−7^, *p = *2.2 × 10^−16^ by the proportion test).

As shown in Figure 6, the protanomalous and trichromatic macaques could discriminate the patterns in more than 90% of the baseline trials. The performance levels in the probe trials were also high and were not significantly different from the baseline trials (proportion test, *p* = .81 and *p = *1 for trichromats, *p = *.78 and *p = *1 for protanomalous). The results indicate that the protanomalous female and trichromatic macaques passed the color-blind test. In contrast, although the dichromatic macaques chose S+ in more than 90% of the baseline trials, they chose the probe as if randomly in the probe trials. Their performance levels in the probe trials were significantly different from those in the baseline trials (proportion test, *p* = 5.7 × 10^−7^, *p* = 2.2 × 10^−16^), but were not different from the chance level (binomial test, *p* = .48, *p* = 1, respectively). These results indicate that dichromatic macaques could not discriminate between S+ and S− and failed to pass the color-blind test. This experiment shows that the protanomalous female had an advantage over dichromatic macaques in discriminating colors, just like trichromatic macaques. Reaction time was not measured in this experiment.

## Discussion

In this study, we tested the color vision performance of protanomalous females in both dichromatic and trichromatic advantage paradigms and found that the protanomalous female macaques successfully discriminated the target pattern, even when it was concealed by a camouflaging color, same as the dichromatic macaques. The protanomalous female also passed the color-blind test similar to normal trichromatic macaques. Thus, the protanomalous macaques were thought to have the combined ecological advantages of being both trichromats and dichromats.

Due to the rarity of dichromacy in the macaque, our data contain only low sample numbers of the protanomalous female. Although there were sex differences between the phenotypic categories, both detection performance and reaction time supported the earlier conclusion.

### Population of L and M Cones in the Retina, Stochastic Event

Genetically, the unique color perception of protanomalous female macaques is based on an extra mutant *L4M5* gene in one of their X-chromosomes. In females, early in embryonic development, one of the two X-chromosomes in any given cone will be randomly inactivated (Lyonization process; Lyon, 1962), which would lead to a decrease in the number of L opsins as well as in the L-M cone ratio compared to that in normal trichromats. Miyahara, Pokorny, Smith, Baron, and Baron (1998) indicated that females heterozygous for the anomalous opsin have a relatively small number of L cones than M cones while they show trichromacy. Hofer, Carroll, Neitz, Neitz, and William (2005) directly measured the relative number of cone mosaics in the heterozygous female using adaptive-optics imaging and found the ratio of L to M cones to be 0.37:1. This may also have happened in our protanomalous macaque. The unique ratio might make the color differences less vivid. This reduction in red-green color signals may make the color camouflage ineffective for the protanomalous female. The signal in the L-M pathway should be reduced but not lost, perhaps increasing the protanomalous female discrimination threshold for red-green differences (Bimler & Kirkland, 2009). On the other hand, although the sensitivity of the red-green channel was decreased, it was sufficient to pass the red-green color-blind test.

### Population of Dichromats in the Wild

Although dichromats have advantages in breaking the color camouflage, their selection in nature is limited compared to the advantages of trichromacy. The current findings of the advantages to the protanomalous female in both breaking color camouflage and trichromacy presume to increase the number of protanomalous females in the population, and thus, to increase the probability of dichromatic individuals. This leads to the idea of an independent advantage conferred by each vision, such as that shown in New World monkey populations composed of allelic trichromats and habitual dichromats (Caine & Mundy, 2000; Melin et al., 2009; Riba-Hernandez et al., 2004; Smith et al., 2003). However, only a few protanomalous females and dichromats exist in the population in Pangandaran, Indonesia, and a few cases of multiple *M* opsin genes exist in southeast Thailand (Onishi et al., 2002). We may interpret this rarity as constrained by the comparative advantage of trichromacy over dichromacy; that is, the ability to break the color camouflage is much less advantageous compared to that of seeing more hues. Increased attention to luminance edges at the expense of chromatic edges can penetrate some forms of camouflage; however, it may also weaken scene segregation based on color signals, especially in a situation with irregular shadows.

### Future Directions

The advantages shown by the protanomalous female imply that the existence of S, M, L, and the additional L4M5 opsin affects color perception in individuals who carry it. Thus, these results may support the assumption regarding the plasticity of the mammalian brain, which enables extraction and comparison of a new dimension of sensory inputs (Jacobs, Williams, Cahill, & Nathans, 2007; Mancuso et al., 2009). However, in protanomalous females, how such additional signals would be aligned into the retinal pathway to affect perception remains uncertain. If a cone cell expressing the hybrid opsin is treated no differentially from an M-cone, it might induce an additional dimension of color vision, such as tetrachromacy in a human female heterozygous for the anomalous opsin (Jordan, Deeb, Bosten, & Mollon, 2010). Although our data have no implications for tetrachromacy in macaques, our protanomalous individuals provide a model to study, in more detail, the retinal and cerebral processing of color vision.

## Conclusion

Our experiments showed that protanomalous female monkeys can break color camouflage similar to protanopic dichromats. Unlike dichromats, they also have an advantage in perceiving the same colors as those shown by trichromats. These perceptions can be explained by the change in the ratio of long- to middle-wave sensitive cones due to the expression of the *L4M5* hybrid gene. The plasticity of the retinal circuit should allow integration of the L4M5 opsin to produce a unique color perception in protanomalous female monkeys.
